# Symmetrical peripheral gangrene complicating ventricular pseudoaneurysm: a report of an unusual case and a brief review of the literature*

**DOI:** 10.1590/abd1806-4841.20165061

**Published:** 2016

**Authors:** Sudip Kumar Ghosh, Biswajit Majumder, Sandip Ghosh, Sharmistha Chatterjee, Megha Agarwal

**Affiliations:** 1 R.G.Kar Medical College – Kolkata, India; 2 College of Medicine and Sagor Dutta Hospital – Kolkata, India

**Keywords:** Blood coagulation disorders, Gangrene, Heart rupture

## Abstract

Symmetrical peripheral gangrene is an ischemic necrosis simultaneously involving the distal portions of two or more extremities without any proximal arterial obstruction or vasculitis. It may occur as a result of a large number of infectious and non-infectious causes. A few cases of symmetrical peripheral gangrene associated with cardiac disease have been described in the literature. We describe a case of symmetrical peripheral gangrene complicating ventricular pseudoaneurysm, probably a hitherto unreported occurrence. In this report, we sought to emphasize the importance of cardiac evaluation while dealing with a case of symmetrical peripheral gangrene.

## INTRODUCTION

Symmetrical peripheral gangrene (SPG) is an ischemic necrosis simultaneously involving the distal portions of two or more extremities without any proximal arterial obstruction or vasculitis.^1-4^ Although the exact etiopathogenesis of SPG remains elusive, a lowflow state is commonly present in association with a hypercoagulable vasospastic situation leading to microcirculatory occlusion. A more or less prototypical clinical presentation of SPG – in spite of a large number of etiological associations – is suggestive of disseminated intravascular coagulation (DIC) as the final common pathway of its pathogenesis.^2,3,4^ We describe a case of symmetrical peripheral gangrene complicating a left ventricular pseudoaneurysm for its unusual and interesting features.

## CASE REPORT

A 54-year-old woman presented to us with progressive breathlessness and gradually progressive, persistent bluish discoloration of the feet and fingers for the preceding 4 days. She had a history of inferior wall myocardial infarction (MI) three months back. Initial examination revealed bluish discoloration and marked coldness of both hands and feet.

**Figure 1:**
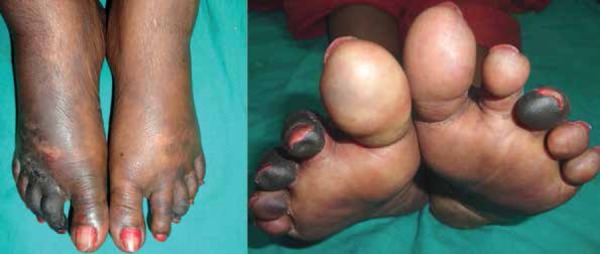
Symmetrical gangrenous changes of both feet

**Figure 2:**
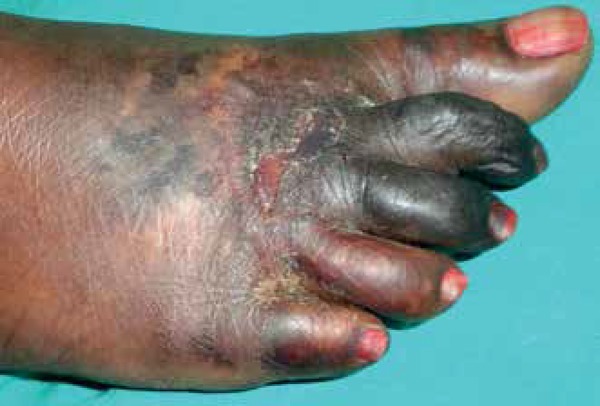
Gangrenous changes, erosions, and purpuric macules

Subsequently, frank gangrene associated with mummification of the toes was observed. (Figures 1 and 2). The patient presented with areas of skin necrosis, purpura, and erosions on the dorsum of the feet and hands. Her blood pressure was low (80/60mm Hg); pulse rate was 110/minute and regular. All peripheral pulses were normally palpable. Jugular venous pulse was not raised. The first heart sound was soft, the second heart sound was normal, and the third heart sound presented a gallop rhythm. We also heard bibasilar fine rales on her chest. Examination of the other systems was noncontributory. Laboratory investigations revealed anemia (hemoglobin: 8.2gram %), mild leukocytosis (12,000/cmm; neutrophil 72%), and low platelet count (70,000/cmm).

C-reactive protein was elevated. Blood urea and serum creatinine levels were normal. Blood lactate level was elevated (3.1 mmol/L). Liver function test showed slightly raised bilirubin (1.5mg/dl) with normal transaminase levels. Lipid profile and serum electrolytes were normal. Bacterial culture of urine, blood, and sputum revealed no organism growth. Screening for hepatitis A, B, C, and HIV was negative. Antinuclear antibody (Hep-2 cell line method) and ANCA were negative. D-dimer assay was positive (400ng/dl). Abdominal ultrasonography was normal. Chest X-ray showed cardiomegaly with pulmonary edema. ECG revealed old inferior wall infarction.

**Figure 3:**
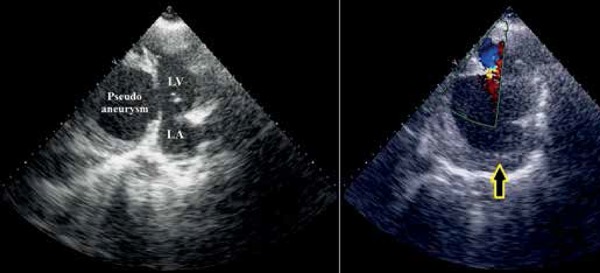
**A, B:** Transthoracic echocardiography showing large submitral pseudoaneurysm with large layered thrombi within it (arrow). (LA= left atrium; A B LV=left ventricle)

Transthoracic echocardiography showed large submitral pseudoaneurysm with large layered thrombi within it and left ventricular systolic dysfunction (Figure 3). The neck of the aneurysm was narrow and the ejection fraction was 25%. Cardiac angiography revealed significant lesion in the ramus intermedius and obtuse marginal arteries.

Peripheral doppler showed no significant blockage in the major lower or upper limb arteries. Although peripheral angiography showed complete patency of the lower limb arteries, slow flow to the distal part was reported. Lesional skin biopsy showed non-specific inflammatory changes. Based on these features, we diagnosed symmetrical peripheral gangrene complicating ventricular pseudoaneurysm. The patient was treated conservatively with supportive treatment, but died on the 5th day after admission to the hospital.

## DISCUSSION

Gangrenous changes in symmetrical peripheral gangrene (SPG) may result from low cardiac output. Consequently, significant reflex vasoconstriction in extremities and splanchnic vessels takes place in order to increase the perfusion of the more vital cerebral and coronary circulations.^1,5^

The basic structural instability of small blood vessels – due to their smooth muscle wall – allows intense vasoconstriction and non-thrombotic occlusion when intraluminal hydrostatic pressure falls below the critical closing pressure.^1^ Large intravascular thrombi arising from intra or extra-cardiac sites have been reported to cause DIC. DIC has been associated with a wide variety of disorders, with infection, malignancy, trauma and surgery accounting for most cases.^2,3,4^ Severe DIC associated with large ventricular thrombi shortly after acute myocardial infarction (MI) has also been reported in the literature.^6^ Therefore, we suspect that the large thrombi present in the pseudoaneurysm were the cause of DIC in our patient.

Our patient developed ventricular pseudoaneurysm, low cardiac output, and acute left ventricular failure as a result of recent MI and compromised coronary arterial circulation. A review of the literature reveals that left ventricular free wall rupture is a fatal complication of MI.^7^ Rarely, the rupture is contained by an adherent pericardium, creating a pseudoaneurysm.

Differentiation between aneurysm and pseudoaneurysm is still a challenge, especially in clinical cases. Zaffoli *et al.* characterize an aneurysm by the absence of ruptures and integrity of the myocardial wall.^7^ However, posterior-inferior location, dyskinesia of the affected area, and narrow-necked cavity favored the diagnosis of pseudoaneurysm in our patient.

Peripheral angiography showed complete patency of the arteries in the SPG cases previously described. However, the rate of filling and flow through the peripheral arterial system was extremely slow in these patients, as in our case.^8^

We believe that low cardiac output as a result of MI and ventricular pseudoaneurysm in conjunction with DIC was the probable mechanisms responsible for SPG in the present patient. SPG can occur as a consequence of a large number of infectious and non-infectious causes. It has been reported to occur in a few cardiological disorders like myocardial infarction, secondary leiomyosarcoma of the right ventricular wall, ball valve thrombus of the right atrium, peripartum cardiomyopathy, paroxysmal tachycardia, pulmonary embolism, low-output heart-failure, among others.^2,5,9,10^ However, SPG complicating ventricular pseudoaneurysm is probably a hitherto unreported condition. The present case report emphasizes the importance of cardiac evaluation while dealing with symmetrical peripheral gangrene.
